# Cydrasil 3, a curated 16S rRNA gene reference package and web app for cyanobacterial phylogenetic placement

**DOI:** 10.1038/s41597-021-01015-5

**Published:** 2021-09-02

**Authors:** Daniel Roush, Ana Giraldo-Silva, Ferran Garcia-Pichel

**Affiliations:** 1 School of Life Sciences, Arizona State University, 85282 Tempe, Arizona USA; 2 Center for Fundamental and Applied Microbiomics, Biodesign Institute, Arizona State University, 85281 Tempe, Arizona USA

**Keywords:** Phylogeny, Metagenomics, Microbial ecology, Genetic databases

## Abstract

Cyanobacteria are a widespread and important bacterial phylum, responsible for a significant portion of global carbon and nitrogen fixation. Unfortunately, reliable and accurate automated classification of cyanobacterial 16S rRNA gene sequences is muddled by conflicting systematic frameworks, inconsistent taxonomic definitions (including the phylum itself), and database errors. To address this, we introduce Cydrasil 3 (https://www.cydrasil.org), a curated 16S rRNA gene reference package, database, and web application designed to provide a full phylogenetic perspective for cyanobacterial systematics and routine identification. Cydrasil 3 contains over 1300 manually curated sequences longer than 1100 base pairs and can be used for phylogenetic placement or as a reference sequence set for *de novo* phylogenetic reconstructions. The web application (utilizing PaPaRA and EPA-ng) can place thousands of sequences into the reference tree and has detailed instructions on how to analyze results. While the Cydrasil web application offers no taxonomic assignments, it instead provides phylogenetic placement, as well as a searchable database with curation notes and metadata, and a mechanism for community feedback.

## Abstract (summary)

**Table:** 

Measurement(s)	Phylogenetic Analysis • computational phylogenetic analysis • Data Quality
Technology Type(s)	Maximum Likelihood Estimation • digital curation
Sample Characteristic - Organism	Cyanobacteria/Melainabacteria group

Machine-accessible metadata file describing the reported data: 10.6084/m9.figshare.15032181

## Background & Summary

Cyanobacteria are oxygenic photosynthetic microorganisms, widespread in both terrestrial and marine ecosystems^1,2^ that sustain many ecosystem services including carbon^3^ and nitrogen fixation^4^. Their detection informs researchers about the primary productivity potential of an ecosystem^1,3^.

The rise of high-throughput DNA sequencing, development of easy to use analysis pipelines^5,6^ and availability of comprehensive biodiversity databases has simplified molecular microbial surveys, including those focused on abundance and diversity of cyanobacteria. Unfortunately, assigning taxonomy to organisms in a survey remains uncertain due to shortcomings of reference databases. Errors in the two most used databases, Silva^7^ and Greengenes^8^, which are well documented^9–11^, lead to recurrent taxonomic misassignments. Though this uncertainty can be viewed as a trade-off for both efficiency and coverage, it becomes amplified when examining cyanobacteria, due to the complex history of cyanobacterial systematics.

Unlike that of other prokaryotes, cyanobacterial taxonomy is governed by two codes, the International Code of Nomenclature of Prokaryotes^12^ (ICNP) and the International Code of Nomenclature for Algae, fungi, and Plants^13^ (ICN). Much of the early work on cyanobacterial classification utilized botanical traditions, including identifying new species based upon morphology alone^14^. However, recent applications of molecular phylogeny have shown morphology as a very broad and inaccurate trait for systematic work^15,16^. In addition, molecular phylogeny has facilitated and unified the identification process, and assisted in the discovery of new organisms. Many polyphyletic, “bad” taxa, are legacies of the early morphology-based system (i.e., *Microcoleus*^17^), but some of them are of rather recent coinage (i.e., *Leptolyngbya*^18^). In modern amplicon analysis and classification, it is not uncommon for two sequences to be assigned to the same species epithet but be separated by vast phylogenetic distance. This problem stems from the practice of delineating new taxa by sequence alignment with a small set of sequences of known organisms most closely related to the one being studied, instead of examining them in the context of a comprehensive phylogeny that would provide a full picture. More uncertainty yet arises from the recent and contentious proposal for the inclusion of non-phototrophic organisms in the Cyanobacterial phylum.

To overcome taxonomic uncertainty, researchers should optimally move away from approaches based on sequence similarity algorithms and databases with lax criteria for sequence inclusion, and instead, manually curate amplicon sequences after traditional taxonomic assignment, or better, use a comprehensive phylogenetic perspective based upon curated, organism specific databases. Bioinformaticians have already taken steps to alleviate these issues by developing algorithms that use the principle of phylogenetic placement. Phylogenetic placement algorithms represent a phylogenetic-accurate and efficient way to perform classification if done on trusted databases, placing query sequences (like those from an amplicon survey) onto a precalculated reference phylogenetic tree, inferred from a curated set of reference sequences^19^. Maximum-likelihood based programs such as PPLACER^20^, RAxML-EPA^21^, and EPA-ng^22^ take any query sequence (or a set) supplied by the user, along with a predefine reference package (a reference phylogenetic tree and alignment of curated sequences), and generate a placement file (JPLACE) that contains statistically explicit placements onto particular nodes of the reference tree as well as their associated confidence values. Perhaps because of the investment required for the creation of reference packages, or because of the complexity in initial data analysis, phylogenetic placement has not been widely adopted, and yet it constitutes a robust and useful methodology.

Here we present such a reference package, Cydrasil 3, intended for Cyanobacteria and its sibling bacterial clades (Margulisbacteria^23^, Melainabacteria^24^, Saganbacteria^25^, Sericytochromatia^26^). The package (Cydrasil 3) offers a framework to simplify cyanobacterial classification, by providing a comprehensive and curated alignment, phylogeny, database, and web application (available at https://www.cydrasil.org) that a researcher with moderate experience can use to conduct a broad examination of the phylogeny of any cyanobacterial sequence(s) of interest. To aid in interpretation, it also includes both tab-separated and JSON-formatted database files with notes and warnings about potential inconsistencies for every sequence in the reference package. We envision three common use cases for the Cydrasil reference package: provide a “first look” at the phylogenetic location of a given 16S rRNA cyanobacterial sequence (of any length) within the context of a full phylogenetic reconstruction, alleviate researchers need to spend time collecting sequences for *de novo* phylogenetic analysis, and act as a reference package for sequence placement algorithms.

## Methods

### Cydrasil database sequence inclusion criteria and version history

**Fig. 1:**
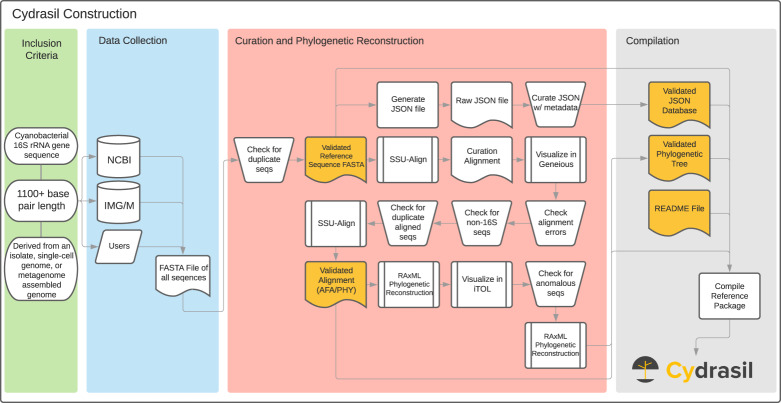
Process flow diagram describing Cydrasil database construction and curation for the version 3 release. Yellow shapes indicate final reference package files. Chart symbols follow American National Standards Institute (ANSI)/International Organization for Standardization (ISO) standards.

From the onset, we implemented strict criteria for sequence inclusion and curation procedures of the Cydrasil database (Fig. 1). Inclusion criteria were as follows:

1. Sequences must come from isolated strains or single-cell genomes. Exception was made for metagenome-assembled genomes after a manual review of the genome This was needed for many representatives of the sibling clades.
2. The minimum sequence length for inclusion was 1100 base pairs. The length was chosen by analyzing all available 16S rRNA gene sequences from the initial data collection and finding a compromise between species coverage and phylogenetic signal (alignment length) that provided a well-supported phylogeny. Of note, this excludes all cyanobacteria sequenced using the Nübel *et al*.^27^ cyanobacteria-specific primers due to amplicon length.

Initial data collection for Cydrasil (and the basis for version 1) included all available cyanobacterial 16S rRNA gene sequences that fit the above criteria and were available through the NCBI taxonomy browser up until June 2016 (Table 1), along with an outgroup comprising the closest known non-phototrophic organisms at the time, *Vampirovibrio spp*. (sister clade Melainabacteria).

**Table 1:** Summary statistics for major releases of Cydrasil.

Cydrasil Version	Number of Sequences				
	Total	Cyanobacteria	Outgroup	Plastids	Sibling clades
1 (rc1)	982	980	0	0	2 (root)
1.5	1494	1481	3	6	4
2	1482	1405	3	6	68
3	**1327**^1^	**1288**	**0**^2^	**5**	**34**

^1^The source distribution of sequences are as follows: 971 NCBI and 356 IMG/JGI.

^2^Outgroup sequences were removed in version 3 as they are no longer needed. The WOR1 sibling clade is now used as root.

After the initial release, we continued to expand the coverage of the Cydrasil database by targeting other data repositories for the next release (version 1.5). We collected all available cyanobacterial 16S rRNA sequences from genomes available on the JGI IMG/M^28^ database up until June 2019 and incorporated the first set of researcher-submitted sequences (Table 1). This release also included six plastid sequences that were added because of user feedback. After the first release, we received feedback from users who were running into issues with certain placements in amplicon surveys from biological soil crusts. Some abundant cyanobacteria sequences, classified as cyanobacteria but not chloroplasts using the GreenGenes database (a recommended initial filtering step when using Cydrasil), were not being accurately placed into the Cydrasil reference tree. A BLAST analysis using the NCBI nr database found that some of the GreenGenes reference sequences were misidentified chloroplasts. The addition of plastid sequences into the Cydrasil reference tree alleviated these ambiguous placements and allowed for the users to confidently place the original sequences. Additionally, a more phylogenetically distant outgroup was also added in preparation for an expansion of the sibling clade representation in a future release.

Cydrasil 2 was an expansion of the v1.5 release and included additional curation measures such as removal of duplicate aligned sequences that only differed by length (longest sequence kept) and a phylogeny-guided anomalous sequence flag and/or removal, which resulted in a reduction of the total number of Cyanobacteria sequences from the previous release (described below, Table 1). For the reference package (sequence list, alignment, phylogenetic tree), we also incorporated all available 16S rRNA sequences from the sibling clades (Margulisbacteria^23^, Melainabacteria^24^, Saganbacteria^25^, Sericytochromatia^26^) that were available through the NCBI taxonomy browser in August 2019. Since version 2 also coincided with the release of the Cydrasil website and web application, we created a JSON-formatted database file (searchable on the Cydrasil website; also included in both data repositories) that included metadata for every sequence.

Cydrasil 3 builds upon the extensive curation changes introduced into version 2, along with an additional complete manual curation of the database itself, entry format changes, and the addition of new community-suggested genera. All sequence names are now formatted in such a way as to promote automated parsing:

CY-sourceName-sourceDatabaseID#g__generaName.s__speciesName.str__strainName

All sequences have been updated to the most recent NCBI taxonomy as of May 2021. Sequences that underwent taxonomic changes either had their names updated or were removed if the new taxonomy was ambiguous (as in the case with almost all representatives of the *Sericytochromatia* sibling clade). Next, to reduce computational overhead and sequence redundancy, we removed highly clustered sequences from overrepresented species and strains. Most of the sequences were those from single-cell sequencing experiments, and as such, led to oversampling of specific strains in the *Prochlorococcus*, S*ynechococcus*, and *Microcystis* genera. Other duplicate strain-level entries were also removed, with the longest sequence taking priority. We removed the remaining “outgroup” sequences (corresponding to *Escherichia coli*, *Listeria monocytogenes*, and *Geobacter sulfurreducens*) as the WOR1 sibling clade provided a more phylogenetically sound root. This in total led to a removal of 202 sequences compared to version 2 of the database. User submission of 47 new sequences put the total number of sequences of Cydrasil version 3 at 1327 (Table 1).

### Data curation and phylogenetic reconstruction

The post-data collection curation procedure for each release began with a global check of the reference sequence file for header or sequence duplication. Duplicate sequences were first removed. Next, to identify duplicate aligned sequences, a curation alignment was generated using SSU-Align 0.1.1^29^ with default parameters and masked using the ssu-mask feature of SSU-Align with per-alignment calculated masks, which removes alignment insert columns and those columns that aligned with low confidence (posterior probability <0.95). The curation alignment was then manually examined in Geneious version 8^30^ for duplicate aligned sequences, non-16S rRNA sequences, and alignment errors. Sequences that were found to be identical post-masking were combined (one sequence was kept, and a note was made in the database file) to reduce database redundancy and computational overhead. Sequences that were poorly aligned (typically due to the unlabeled inclusion of the ITS and/or 23S regions) were trimmed and removed if their length fell below the 1100 base pair threshold. Alignment-based curation for all Cydrasil versions 1 (rc1) and 1.5 ended here with a validated reference sequence file and the generation of a final “validated alignment.”

For Cydrasil 2 and all future releases (including version 3), an additional alignment curation step was added. Due to the introduction of 440 JGI IMG/M genome 16S rRNA sequences in v1.5, some organisms had both an NCBI sequence and an IMG/M sequence, or even multiple IMG/M sequences. These sequences were kept due to the previous curation protocol indicating that the sequences were unique. However, upon closer examination, some entries were found to be the exact same sequence, with the only difference being sequence overhang on the 5′ and/or 3′ end. The inclusion of both the parent and child sequences had little effect on the final reference alignment and phylogenetic tree, but for reduction of computational overhead and user readability, the longest sequence was kept, and the children sequences were removed. Then, a final “validated alignment” was generated.

After the alignment curation step, each release then underwent the same phylogenetic tree-based curation procedure. In the case of Cydrasil 3, the validated alignment was used as the input for a full maximum likelihood phylogenetic reconstruction using RAxML-NG^31^ in the RAxML-NG (1.0.1) on XSEDE, part of the CIPRES^32^ science gateway. The run included combined tree search and bootstrapping analysis (--all) using the autoMRE bootstrapping convergence test^33^ and a SYM + G4 model determined using modeltest-NG^34^. The output curation phylogenetic tree was examined using iTOL v6^35^ for inconsistencies in taxonomic groupings, anomalous phenotypic clustering, lone wolf sequences, and sequences that directly contradict widely accepted theories regarding the evolution of Cyanobacteria. If a sequence fit any of these criteria, a literature search was conducted to identify possible causes for the abnormality. Typically, the erroneous sequences were removed, but in special cases where the organism was the type species for a genus or in common databases for taxonomic assignment, the sequence was kept with a clear warning in the header. This warning was also included in the Cydrasil 3 database file. Once all anomalous sequences were removed, the tree was re-run and marked as the validated phylogenetic tree.

## Data Records

The Cydrasil reference dataset contains a reference sequence list (FASTA), alignment files (FASTA and PHYLIP), a SSU-Align masking file (.mask), a RAxML-NG model file (.bestModel), a phylogenetic tree (NEWICK), database files (JSON and TSV), and a README file (in Markdown) that includes instructions for using the reference database for sequence placement and data analysis using iTOL. All files are deposited in figshare (10.6084/m9.figshare.c.5446053)^36^ and Zenodo (10.5281/zenodo.4885039)^37,38^.

The reference sequence list is a standard FASTA file with a header name that either includes the IMG gene id or the NCBI accession number, and the NCBI taxonomy associated with the sequence. The header name has been formatted to be easily parsable using automated methods and is compatible with NEWICK format limitations.

The Cydrasil alignment is provided in both FASTA and relaxed PHYLIP formats. This allows for the user to use various popular algorithms for aligning query sequences to the Cydrasil reference alignment. We have also included the SSU-Align mask file that was generated during reference alignment construction, in the case the user desires to use SSU-Align or Infernal^38^ for query sequence alignment.

An unrooted tree file is included in the dataset for use with sequence placement algorithms. A tree model parameter file has also been included for use with epa-ng for sequence placement.

Database files (in JSON and TSV formats) contain the sequence and metadata fields to provide the user with basic information about the organism and a link to the data in its respective database. An overview of the metadata fields for the JSON-formatted file is described in Table 2. The same fields are column headers in the TSV database file.

**Table 2:** JSON keys, and description for cydrasil-v3.json. All entries are strings.

*JSON Key*	Description
*cyID*	New v3 sequence ID in CY-dataSource-dataSourceID format
*cyTaxID*	New v3 taxonomic ID in the CY-sourceName-sourceDatabaseID#g__generaName.s__speciesName.str__strainName
*dataSource*	Database, publication, or lab where the sequence was retrieved.
*dataSourceLink*	A link to the entry in the corresponding dataSource or contact information for the submitting lab.
*id*	The id number of the sequence, initially assigned alphabetically.
*notes*	Contains notes about sequences including other names if strains are identical, or if the organism is part of an outgroup.
*ogCyName*	Name corresponding to the sequence in Cydrasil version 2 and earlier.
*sequence*	The DNA sequence corresponding to the 16S rRNA gene with no masking.
*warnings*	This is reserved for warnings regarding sequence quality, taxonomic naming errors, or other oddities.

Each release also contains a README file (in Markdown format) that includes instructions and tips for using the Cydrasil reference package. The file contains step-by-step instructions on using Cydrasil on a local computer, links to the Cydrasil web application and a visualization of the phylogenetic tree, instructions on how to interpret results, and contact information.

## Technical Validation

Database construction was entirely based upon a manual search and download of sequences from either NCBI or JGI IMG/M. Each entry was manually verified to fit the inclusion criteria before going through the extensive curation process described in the Methods. In the case of researcher submitted sequences, each sequence was manually checked for fidelity and then the submitter contacted for verification of the inclusion criteria. All sequences contain their original header names. Every sequence in the database has dataSource and dataSourceLink information to allow for the end user to verify the original source of the sequence.

## Usage Notes

The Cydrasil reference database and web application (available at https://www.cydrasil.org) is provided as a free public resource for researchers conducting phylogenetic analyses of cyanobacteria. We encourage any researcher looking to identify a new isolate or those conducting amplicon surveys to examine their data in the context of the full Cydrasil 3 phylogenetic reconstruction.

The construction of the Cydrasil 3 reference dataset allows for multiple use cases. The two most common (a quick check of a single sequence or an analysis of a cyanobacterial prefiltered amplicon survey result) are both based on the same sequence placement bioinformatic pipeline designed to place sequences (originally short reads, ranging from 220 to 400 base pairs, from amplicon surveys, but long sequences work as well) onto the branches of a reference tree without modifying the topology. The sequence placement algorithms at the heart of this workflow, like EPA-ng and PPLACER, require a comprehensive reference package that is typically time consuming to create and curate. Cydrasil solves this problem for cyanobacterial research. We designed a user-friendly web application to simplify the workflow so a user could obtain a “first look” at the phylogenetic location of a given 16S rRNA cyanobacterial sequence or analyze a full amplicon survey without the need to install any programs locally. Importantly, the web app does not give any taxonomic assignments, but rather provides a framework for the user to examine a sequence of interest within a phylogenetic context and use the provided NCBI taxonomy of the reference tree only as a frame of reference. The Cydrasil web application has a free, user-friendly sequence placement pipeline based on PaPaRa^39^ and EPA-ng with in-depth instructions on how to analyze the output using iTOL. In the case of a full amplicon survey, the app can scale to thousands of 16S rRNA sequences and includes instructions on how to prepare the output of Qiime2^5^ for use with Cydrasil. If a user wants to use the Cydrasil 3 database for sequence placement locally, an in-depth README file containing detailed instructions is available on the Cydrasil website and available in the figshare, Zenodo, and GitHub repositories.

A third use case is to use the reference dataset as a framework for *de novo* phylogenetic reconstruction of novel long to full length sequences. A user would first conduct an exploratory analysis using the sequence placement pipeline. Then, they would retrieve sequences from the database where the query sequence was placed, along with sequences belonging to the nearest neighbors and a small collection of phylogenetically close, but unrelated sequences to act as an outgroup. With the addition of other high similarity sequences from NCBI (found using a simple BLAST^40^ search), a user could then generate and alignment and conduct a full *de novo* phylogenetic analysis.

Important to the underlying design of Cydrasil is the use of community feedback for future updates. Cydrasil is intended as a “living” reference package, that grows and expands with researchers’ needs. We invite all users of Cydrasil to suggest possible new clades for inclusion, and additionally, submit new sequences to be incorporated in the next release. Cydrasil is under continuous development, and we intend on Cydrasil being a mainstay in cyanobacterial systematics moving forward.

## Data Availability

The source code for the web app is available at https://github.com/droush/cydrasil-web-app. Current and in development versions of the reference package are available at https://github.com/FGPLab/cydrasil. Scripts used in the generation of the reference package and a Markdown file describing the phylogenetic pipeline used are available at https://github.com/FGPLab/cydrasil-helper.
